# The Diagnostic Accuracy of One-Step Nucleic Acid Amplification for Lymph Node Metastases of Papillary Thyroid Carcinoma： A Systematic Review and Meta-Analysis

**DOI:** 10.3389/fendo.2021.757766

**Published:** 2022-01-04

**Authors:** Xiaofei Wang, Xun Zheng, Jingqiang Zhu, Zhihui Li, Tao Wei

**Affiliations:** Department of Thyroid & Parathyroid Surgery, Laboratory of Thyroid and Parathyroid Disease, Frontiers Science Center for Disease-Related Molecular Network, West China Hospital, Sichuan University, Chengdu, China

**Keywords:** papillary thyroid carcinoma, diagnosis, one-step nucleic acid amplification (OSNA), lymph node metastases, meta-analysis

## Abstract

**Background:**

One-step nucleic acid amplification (OSNA) analysis is a molecular diagnostic technique for lymph node metastases (LNMs) by quantifying cytokeratin 19(CK 19) mRNA. We aim to evaluate the intraoperative diagnostic accuracy of OSNA assay for LNMs of papillary thyroid carcinoma (PTC).

**Methods:**

PubMed, Embase, Cochrane Library, and Web of Science databases were searched to retrieve related literature. A meta-analysis was performed using STATA11.0, Meta-Disc 1.4 and RevMan 5.3.

**Results:**

This meta-analysis included six studies involving 987 lymph nodes from 194 patients. The pooled sensitivity, specificity, and area under the summary receiver-operating characteristic curve (AUC) of OSNA for detecting LNM were 0.88, 0.90, and 0.95, respectively.

**Conclusion:**

OSNA assay is an accurate molecular diagnosis for intraoperative detection of lymph node metastasis in PTC.

## Introduction

Papillary thyroid carcinoma (PTC) is the most common endocrine-related malignances. Although PTC has an excellent prognosis, its incidence of central compartment lymph node metastases (LNM) is relatively high, reported to be 20-90% (1–3). However, whether the surgical treatment of PTC requires central neck compartment lymph node dissection (LND) is a controversial topic because of the potential risks of hypocalcemia and recurrent laryngeal nerve injury and the doubtful prognostic benefits (4–7). The American Thyroid Association (ATA) recommends that therapeutic central compartment LND should be performed when LNM has been confirmed before or during surgery, and prophylactic LND should be performed in high-risk patients with stages T3 and T4 of the TNM classification (8). Therefore, accurate identification of neck LNM is essential for selecting the appropriate surgical plan for the treatment of PTC patients.

Ultrasonography has been routinely used for preoperative evaluation of PTC patients because of its high sensitivity in detecting cervical LNM. However, it has poor accuracy for central lymph node compartment, the most frequent site of lymph node metastasis of PTC. According to reports, occult LNM was detected by pathological examination in up to 50% of patients who had no clinical evidence of LNM (cN0) on preoperative imaging (7, 9). As other tumors, selective sentinel lymph node (SLN) biopsy has been applied in patients with PTC to improve the diagnostic performance of LNM (10, 11). SLN biopsy can reduce the risk of complications associated with unnecessary LND and avoid a potentially more difficult reoperation for occult metastatic lymph nodes. Intraoperative frozen section examination was the common method to evaluate the status of SLN. However, the false negative rate of intraoperative frozen section examination is as high as 23.3% (12), and the specificity in detecting micrometastases is limited (13). Therefore, a more accurate and quick intraoperative diagnostic technique is urgently needed.

One-step nucleic acid amplification (OSNA) is a rapid and precise molecular diagnostic method based on reverse transcription loop-mediated isothermal amplification of cytokeratin 19 (CK19) mRNA. Recently, it has been introduced for intraoperative analysis of lymph node involvement in many tumors such as breast cancer (14), colorectal cancer (15), lung cancer (16), and endometrial cancer (17). It is known that about 70% of all PTCs have an intense CK19 expression at the membrane level, and that positive expression can also be seen in metastatic cells within the lymph nodes (18). Non-metastatic cells will not express CK19. This leads to the consideration of OSNA in this case. Several recent studies have investigated the application of OSNA in PTC (19–24). The purpose of this systematic review and meta-analysis was to evaluate the efficacy of OSNA versus routine histopathology in the intraoperative detection of LNM in PTC.

## Methods

This review was conducted in accordance with the guidelines by the Preferred Reporting Items for Systematic Review and Meta-Analyses (PRISMA) (25).

### Search Strategy

Four databases (PubMed, Embase, Cochrane Library, Web of Science) were systematically searched for all relevant studies regarding the diagnostic accuracy of OSNA published from January 2006 to January 2021. The following search terms were used: “one-step nucleic acid amplification” or OSNA and “papillary thyroid carcinoma” or “papillary thyroid cancer”. The references of all the eligible papers and reviews articles were screened for further studies.

### Inclusion and Exclusion Criteria

The inclusion criteria were as follows: (1) patients included in studies had a confirmed pathologic diagnosis of PTC; (2) evaluating the diagnostic accuracy of OSNA for LNM; (3) postoperative hematoxylin-eosin (HE) staining was used as the gold standard in lymph node evaluation; (4) providing sufficient information for calculating values of true-positive (Tp), false-positive (Fp), false-negative (Fn), and true-negative (Tn). Review articles, conference abstracts, letters, case reports, comments, or non-English literatures were excluded.

### Data Extraction and Quality Assessment

Data extraction and quality assessment for each included study were conducted independently by two reviewers (XF. Wang and M. Yang). Disagreements are resolved through discussion. Extracted information includes first author, year of publication, country, study design, number of patients, number of lymph nodes, reference standard method, whether immunohistochemistry (IHC) confirmed the expression of CK19 in tumor cell after fine-needle aspiration biopsy (FNAB) examination of the tumor before surgery, number of Tp, Fp, Fn, and Tn, and discordant node results.

The quality of the included studies was assessed using the tool for Quality Assessment of Diagnostic Accuracy Studies (QUADAS-2) (26). This tool comprises four key domains: patient selection, index test, reference standard, and flow and timing. For each domain, the risk of bias and applicability concerns (the latter not applying to the flow and timing domains) were analyzed and rated as low risk, high risk and unclear risk.

### Statistical Analysis

Since threshold effect is an important source of heterogeneity, it was first evaluated based on Spearman’s correlation coefficient. If the P-value was more than 0.05, there was no threshold effect. Heterogeneity caused by non-threshold effect was further evaluated by Cochran- Q test and I-square statistic. When P<0.05 or I>50%, which meant existence of significant heterogeneity, a random-effect model was performed to calculate the pooled sensitivity, specificity, positive likelihood ratio (PLR), negative likelihood ratio (NLR), and diagnostic odds ratio (DOR); otherwise, a fixed effects model was used. The performance of OSNA over routine histology was assessed by the area under the curve (sAUC) generated by the summary receiver operator curve (SROC). Publication bias was evaluated by Deeks’ funnel plot asymmetry test. Statistical software package STATA11.0 (StataCorp, College Station, TX, USA), Meta-Disc 1.4 (Unit of Clinical Biostatistics, Ramo e Cajal Hospital, Madrid, Spain), and RevMan 5.3 (Revman, the Cochrane Collaboration) were used for statistical analyses.

## Results

### Study Selection

**Figure 1:**
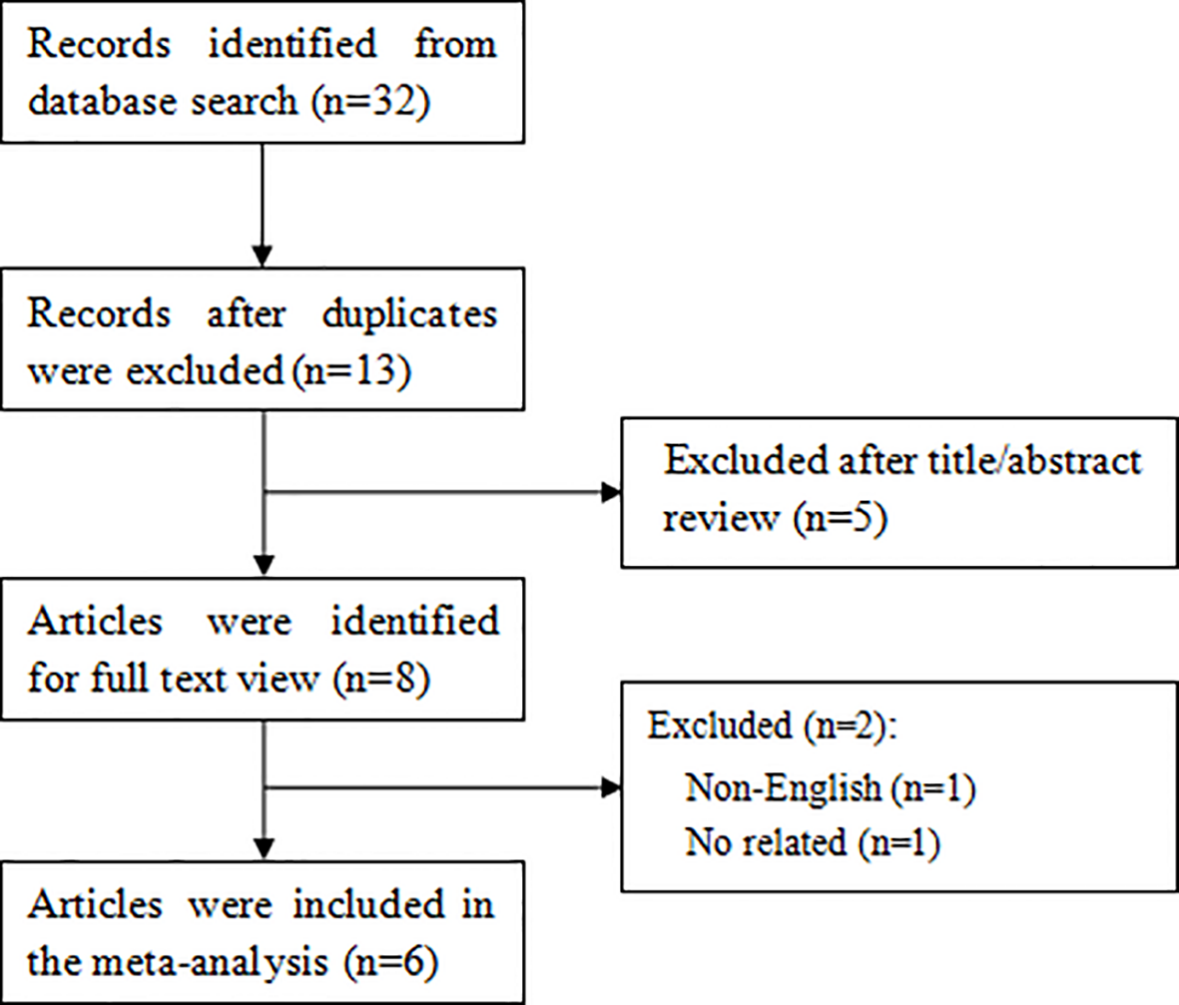
Flow diagram of the study selection process.

As shown in **Figure 1**, a total of 32 articles were retrieved through the search strategy. Of these, 19 duplicate studies were excluded and then 5 studies were excluded after screening the titles or abstracts. After further reviewing the full text of the remaining 8 articles, 6 articles (19–24) finally satisfied the eligibility criteria.

### Study Characteristics

All the included studies were published between 2014 and 2019 and contained a total of 987 lymph nodes from 194 patients. All studies compared histological examination with OSNA in detecting LNM. However, the lymph node workup for pathological and OSNA examination was different. In four studies (20, 21, 23, 24), each lymph node was divided into two halves from the center, the first one for histopathological analysis and the second for OSNA assay. In another study (19), lymph node was divided into four blocks, and two discrete quarters were used for OSNA and histopathology. In the remaining study (22), lymph node was cut into blocks at 2mm intervals, and nonadjacent blocks were subjected to either the OSNA analysis or histological examination. The detailed pathological examinations were also different in all studies. Two studies (21, 24) adopted HE staining and IHC, two (20, 23) adopted HE staining and imprint cytology, and two studies (19, 22) applied HE staining only. The same OSNA system (RD-100i-OSNA, Sysmex Corp., Japan) and the same cut-off point to define positive (≥250 copies/μl) were used in all studies. Discordant rate is present between OSNA and histopathological examination, ranging from 7.6% to 16.2%. The detailed characteristics of the included studies are given in **Table 1**.

**Table 1:** Characteristics of included studies.

Author	Year	Country	No. of patients	No. of nodes	Tp	Fp	Fn	Tn	Reference method	Preoperative CK19 IHC	Discordantnode results(%)
**del Carmen et al.** (21)	2016	Spain	37	284	75	18	14	160	HE/IHC	Yes	32 (11.3)
**González et al.** (20)	2015	Spain	5	50	19	3	2	26	HE/IC	Yes	5 (10.0)
**Medas et al.** (24)	2019	Italy	13	26	7	1	1	17	HE/IHC	Unknown	2 (7.7)
**Iglesias Felip et al.** (23)	2019	Spain	35	470	75	74	2	319	HE/IC	Yes	76 (16.2)
**Kaczka et al.** (19)	2014	Poland	32	92	13	3	4	72	HE	Unknown	7 (7.6)
**Kaczka and Pomorski** (22)	2017	Poland	43	65	17	5	3	40	HE	Unknown	8 (12.3)

Tp, true-positive; Fp, false-positive; Fn, false-negative; Tn, true-negative; HE, hematoxylin-eosin; IHC, immunohistochemistry; IC, imprint cytology.

### Risk of Bias and Quality Assessment

There was no evidence of potential publication bias, with symmetrical appearance on Deek’s funnel plots shape and P value of 0.79 (**Supplementary Figure 1**). The quality of individual study was assessed according to the QUADAS-2. As shown in **Supplementary Figure 2**, the risk of bias was low and the quality of included studies was moderate to high.

### Diagnostic Performance

**Figure 2:**
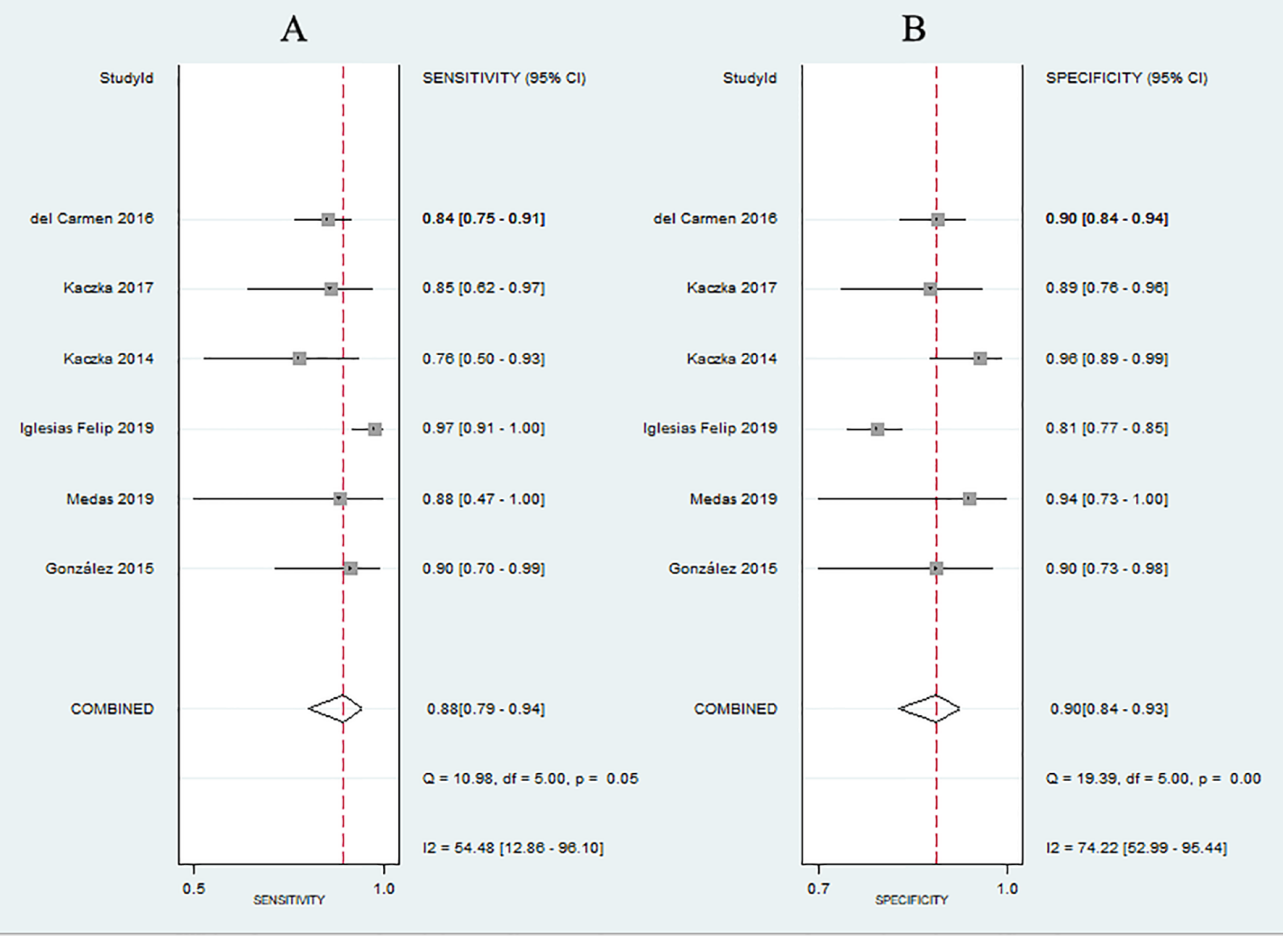
Forest plots of pooled sensitivity and specificity.

**Figure 3:**
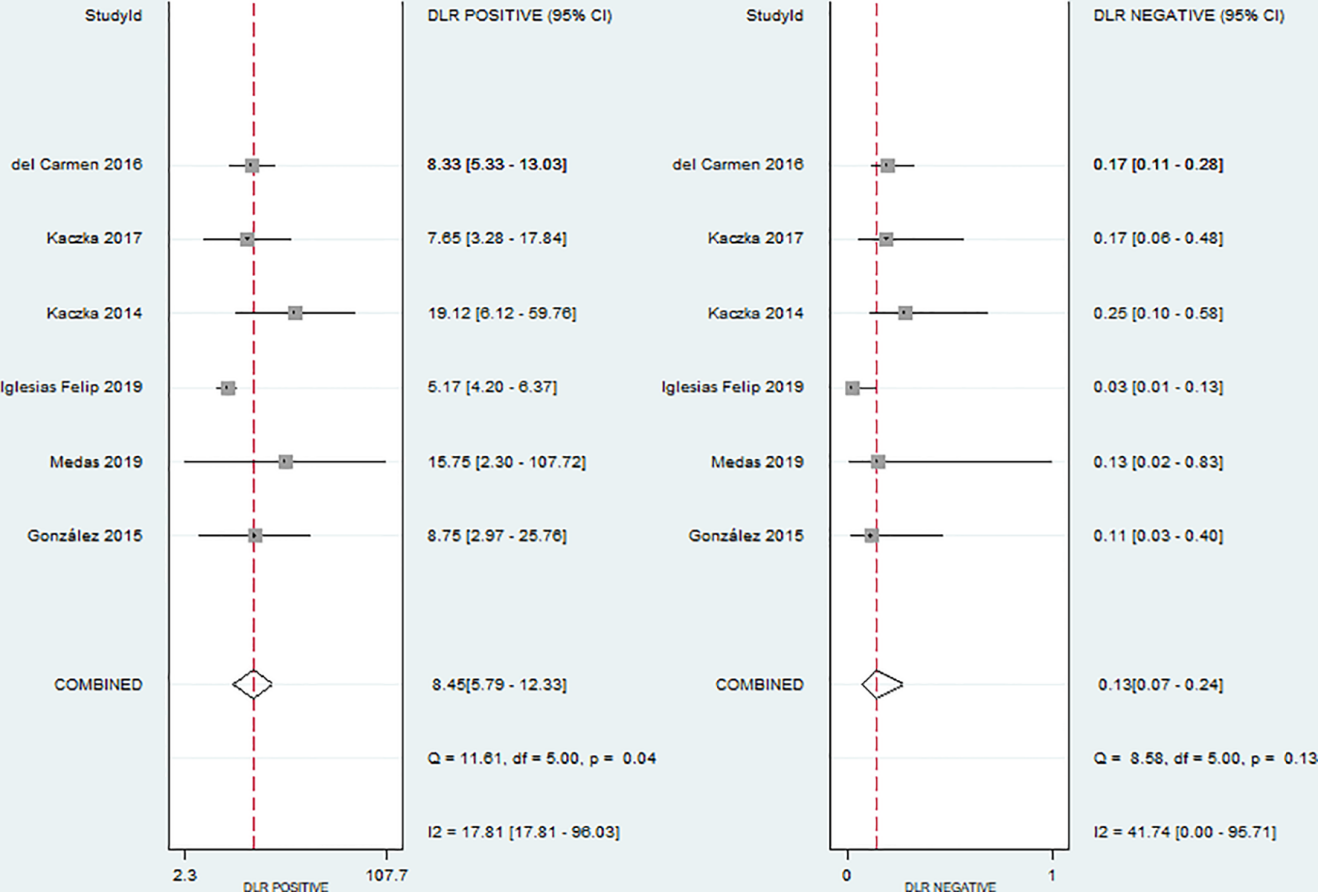
Forest plots of pooled positive likelihood ratio and negative likelihood ratio.

**Figure 4:**
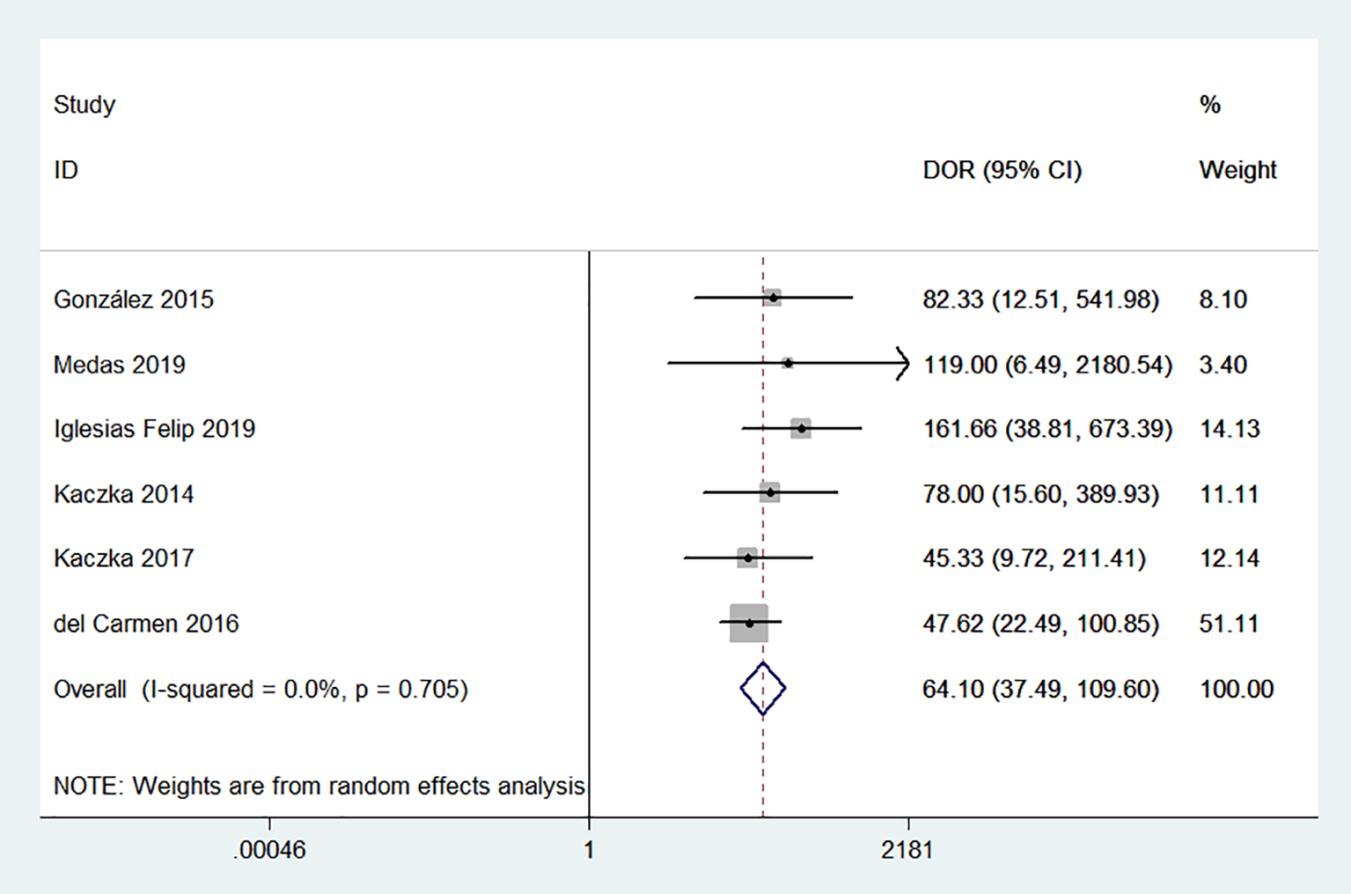
Forest plots of pooled diagnostic odds ratio (DOR).

**Figure 5:**
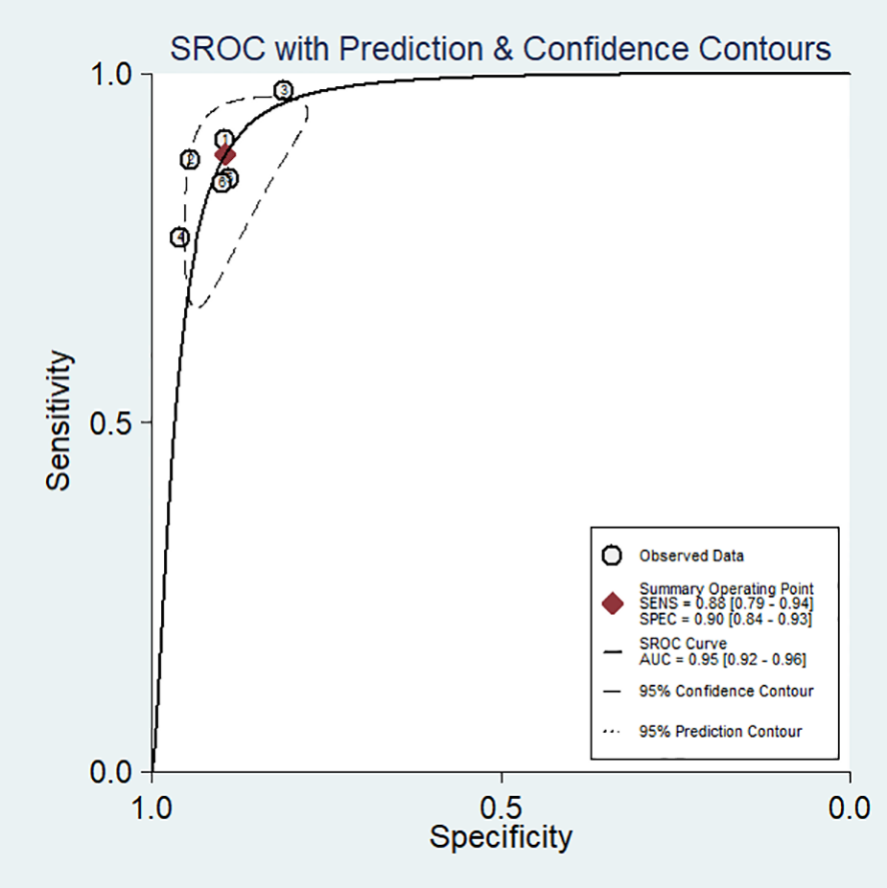
Summary receiver operating characteristic (SROC) curve for the diagnostic accuracy of OSNA assay in detecting lymph node metastases in papillary thyroid cancer.

No evidence of a threshold effect was observed, because the spearman correlation coefficient was -1.00 and P-value was 1.00 in the threshold analysis. However, there was a significant heterogeneity caused by non-threshold effects. As shown in **Figure 2**, the values of *I^2^* for sensitivity and specificity were 54.48% and 74.22%, respectively. Given the significant heterogeneity, a random effect model was used for the meta-analysis. The pooled sensitivity and specificity (**Figure 2**) of OSNA for detecting LNM were 0.88(95% CI 0.79–0.94) and 0.90 (95% CI 0.84–0.93), respectively. The pooled PLR and NLR (**Figure 3**) were 8.45 (95% CI 5.79–12.33) and 0.13 (95% CI 0.07–0.24), respectively. The pooled DOR (**Figure 4**) was 64.10 (95% CI 37.49–109.60) and AUC (**Figure 5**) was 0.95 (95% CI 0.92–0.96), respectively.

## Discussion

OSNA has been widely accepted as a standard tool for evaluating LNM in breast cancer (14, 27, 28). More recently, OSNA has begun to be applied to other tumors (15–17), but so far, its application in PTC has not been widely evaluated. Until recently, some small-sample studies started to evaluated its application in PTC (19–21, 23). In this study, we meta-analyzed six studies that assessed the diagnosis value of OSNA. The results indicated that the pooled sensitivity, specificity, PLR, NLR, DOR and AUC of OSNA assay were 0.88(95% CI 0.79–0.94), 0.90 (95% CI 0.84–0.93), 8.45 (95% CI 5.79–12.33), 0.13 (95% CI 0.07–0.24), 64.10 (95% CI 37.49–109.60) and 0.95 (95% CI 0.92–0.96) respectively. Therefore, it seems that OSNA has a higher accuracy in detecting LNM of PTC. To our knowledge, this is the first meta-analysis to evaluate the diagnostic accuracy of OSNA in PTC.

In fact, because the current evidence on the effect of prophylactic central node LND on the prognosis of PTC patients are controversial, different guidelines on indications for prophylactic central node dissection are not uniform. The ATA guideline recommends that prophylactic LND should be performed in patients with stages T3 and T4 (8). However, prophylactic central node LND was routinely recommended by the Japanese Society of Thyroid Surgeons and Japan Association of Endocrine Surgeons (JSTS/JAES) (29). LND may cause perioperative complications in a certain proportion of patients, such as hypoparathyroidism and recurrent laryngeal nerve injury. Conversely, failure to clean the central lymph nodes leads to the risk of understaging and undertreatment. In this context, SLN assessment seems to be a good way to solve this dilemma. Considering the obvious advantages of patients and surgeons, the use of SLN in PTC has attracted increasing interest (30, 31). Frozen section and/or imprint cytology examination are the most used intraoperative evaluation methods for SLN. However, the false-negative rate of these methods is relatively high, which may be caused by an inevitable random factor in lymph node sampling, specifically if small volume nodes or micrometastases are present. In addition, the low quality of the morphological images provided by the frozen section or imprint cytology may also lead to an error interpretation.

As showed in breast cancer, the OSNA method can overcome these problems. In fact, compared with conventional intraoperative pathological examination, OSNA has obvious advantages: short turnaround time, objective and repeatable. OSNA analysis can evaluate four lymph nodes at the same time, while pathological evaluation can only evaluate one lymph node at once (32). The turnaround time of OSNA to detect one nodule is all less than 40 minutes, which indicates that OSNA can meet the time requirements for rapid diagnosis of lymph nodes and can be incorporated into the intraoperative clinical setting. In addition, the turnaround time can be further shortened with the accumulating experience of technicians. The result of pathological examination depends on the subjective judgment of the pathologist, and the accuracy of the result may be affected by the experience and professional knowledge of pathologists (33). Since OSNA quantitatively evaluates the number of CK19 mRNA copies and the results are related to the number of cancer cells in the nodules, and all OSNA procedures follow a unified protocol, thus the results are objective and repeatable. Our results show that the accuracy is very high (AUC = 0.95) in detecting lymph nodes metastases in PTC, which confirms the reliability of OSNA. Such accuracy seems to be sufficient to support the use of OSNA for PTC. These results are reinforced by the consistency of the methods among the included studies. In all studies, each lymph node underwent both OSNA (with the same threshold of 250 CK19 mRNA copies/μl) and pathologic diagnosis (considered the gold standard).

Despite the high accuracy found, there are still inconsistencies between OSNA results and conventional pathological results. Some reasons might be responsible for these discrepancies. One fact is that metastatic deposits are usually small and irregularly distributed within the lymph node. In all the included studies, half of the nodule was analyzed by OSNA, and the other half was examined by histopathology. Therefore, it is impossible to analyze the same slice using both techniques. It’s likely that, in discordant cases, the metastatic cells may be restricted to half of OSNA or histopathological analysis, respectively. Secondly, CK19 is the only marker used in OSNA assay, metastatic lymph nodes without CK19 expression will be recorded as false-negative nodes by OSNA. Considering that 30% of PTC was CK19-negative (18), it is recommended to perform CK19 IHC on the primary tumor diagnostic fine needle aspiration biopsy (FNAB) samples before using OSNA to assess the lymph nodes of PTC patients. Another possible reason is that ONSA is a quantitative detection technology, and only when the measured value reaches a certain threshold, it will be judged as a positive result. If there are only micrometastases in the lymph nodes (qualitative cytologically judged as positive), quantitative ONSA may be judged as a negative result. It in fact indicates that the OSNA equivalent value is 100-250 CK19 mRNA copies when there are very few metastatic cells in the lymph node (≤0.02 mm according to histological evaluation).

OSNA analysis also has several inherent limitations. OSNA cannot conduct adequate histopathological studies on lymph nodes. For example, it doesn’t provide morphological information on presence of extra-lymph node invasion, which is important information for predicting prognosis (34). Second, OSNA can be only performed when the primary neoplasm is positive for CK-19 expression. Finally, it is also uncertain whether patients upstaged with OSNA over conventional pathological examination will benefit from adjuvant therapy.

There are two limitations in our work. Firstly, statistical heterogeneity of sensitivity and specificity were observed in our study. However, due to the small number of studies included, it is impossible to investigate potential causes of heterogeneity. Secondly, non-English studies were excluded from the meta-analysis, which may lead to selection bias. Thirdly, we cannot evaluate the cost-effectiveness of OSNA for lymph node detection in PTC patients due to the lack of relevant research. For the pathology department, OSNA will come at some costs for equipment, reagents, training and staffing. However, in several studies analyzing the cost-effective of OSNA technique, compared with conventional histopathology for intraoperative diagnosis of SLN metastases in breast cancer patients indicated that OSNA can reduced healthcare costs (35, 36).

In conclusion, our meta-analysis indicates that OSNA assay is an accurate molecular method for intraoperatively detecting LNM in PTC. However, the value of adjuvant therapy in those upstaged by OSNA is uncertain. Moreover, long-term outcomes and rates of disease recurrence with or without OSNA evaluation are unknown. Further studies should be conducted to validate these notions.

## Data Availability Statement

The data are available upon request from the corresponding author (docweitao@sina.com).

## Author Contributions

XW and TW designed the study. XW, XZ, and JZ collected, analyzed data, and wrote the paper. ZL and TW helped in data analysis and manuscript revision. All authors reviewed the manuscript and agree to be accountable for all aspects of the work.

## Funding

This study was supported by Department of Science and Technology of Sichuan Province (2020YFS0166) and Education Department of Sichuan Province (17ZA0170).

## Conflict of Interest

The authors declare that the research was conducted in the absence of any commercial or financial relationships that could be construed as a potential conflict of interest.

## Publisher’s Note

All claims expressed in this article are solely those of the authors and do not necessarily represent those of their affiliated organizations, or those of the publisher, the editors and the reviewers. Any product that may be evaluated in this article, or claim that may be made by its manufacturer, is not guaranteed or endorsed by the publisher.

## Supplementary Material

Supplementary Figure 1 Deeks’ funnel plot for assessing publication bias.

Supplementary Figure 2 Quality evaluation of included studies according to QUADAS-2 **(A)** overall and **(B)** by study.
