# Supplementary figures and images for: The Diagnostic Accuracy of One-Step Nucleic Acid Amplification for Lymph Node Metastases of Papillary Thyroid Carcinoma： A Systematic Review and Meta-Analysis
Source: Front Endocrinol (Lausanne). 2022 Jan 4;12:757766. doi: 10.3389/fendo.2021.757766 (PMC8764176; doi:10.3389/fendo.2021.757766)

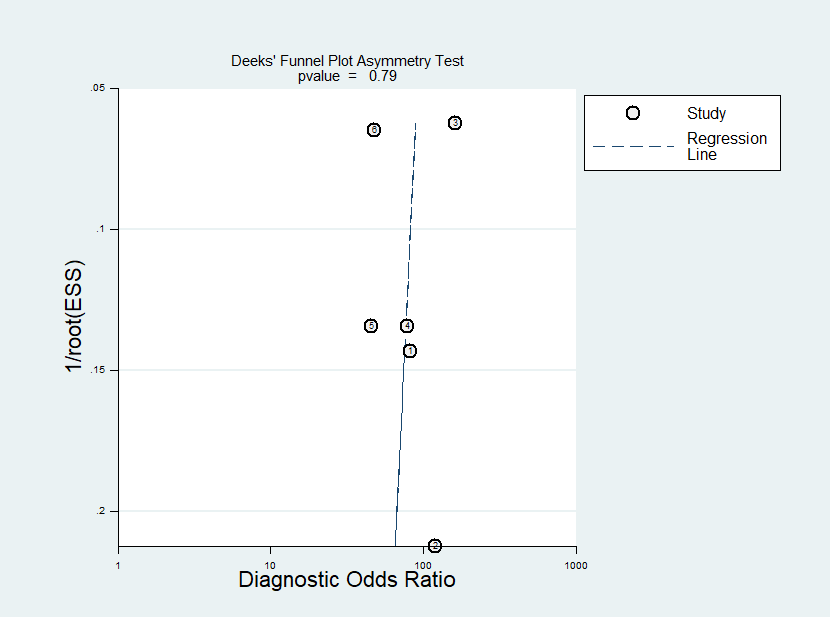

Supplement: Supplementary Figure 1 — Deeks’ funnel plot for assessing publication bias. [file Image_1.tif]

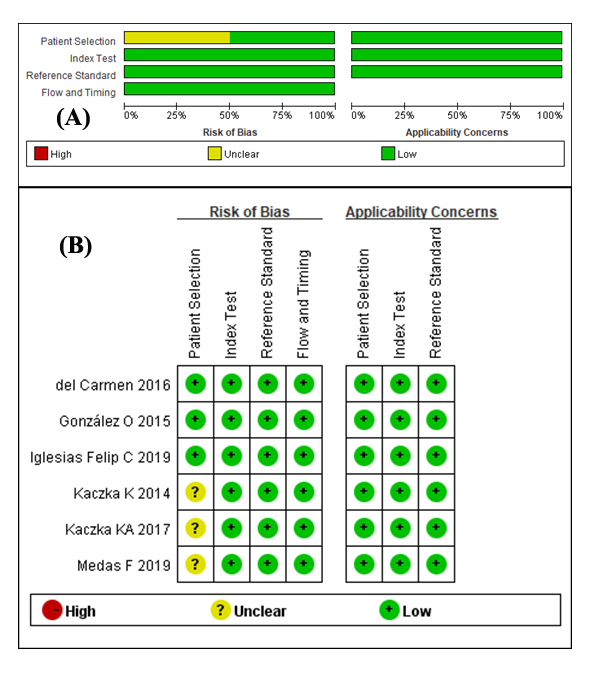

Supplement: Supplementary Figure 2 — Quality evaluation of included studies according to QUADAS-2 (A) overall and (B) by study. [file Image_2.tif]
